# Supplementary material for: Influence of Different Glycoproteins and of the Virion Core on SERINC5 Antiviral Activity
Source: Viruses. 2021 Jun 30;13(7):1279. doi: 10.3390/v13071279 (PMC8310182; doi:10.3390/v13071279)
Supplement: Supplementary file 1 [file viruses-13-01279-s001.zip › viruses-1225048-supplementary.pdf]

Supplementary Material

Table S1. Expression plasmids used in this study.

Glycoprotein	Plasmid	Source	Addgene Number	Reference
ALV EnvA	pCB6-EnvA		74420	[1]
LCMV Arm53b	pCMV WT GP	Juan de la Torre (Scripps Research)		[2]
C-term. truncated SARS CoV S	pKS-SARS-SΔ19	Shuetsh Fukushi (National Institute of Infectious Diseases, Japan)		[3]
M-PMV Env	pTMT	Eric Hunter (Emory Univ.)		[4]
HTLV-1 Env	pV1/HTLV	Paul Bieniasz (Rockefeller Univ.)		[5]
MLV Eco Env	pHCMV-EcoEnv	Miguel Sena-Esteves (Univ. of Mass. Med. School)	15802	[6]
MLV Ampho Env	pHCMV-AmphoEnv	Miguel Sena-Esteves (Univ. of Mass. Med. School)	15799	[6]
HERV-K Env	pCAGGS-HERV-K	Sean Whelan (Harvard Univ.)		[7]
PIV5 F	pCAGGS-PIV5 F	Sean Whelan (Harvard Univ.)		[8]
PIV5 HN	pCAGGS-PIV5 HN	Sean Whelan (Harvard Univ.)		[8]

Flu HA (H7/Kp Rostock)	pFPV-HA	John Olsen (Univ. of North Carolina Chapel Hill [Emeritus])		[9]
Flu NA (H1N1; A/PR/8/34)	pEF6-NA	John Olsen (Univ. of North Carolina Chapel Hill [Emeritus])		[10]
Flu M2	pCB6-M2	John Olsen (Univ. of North Carolina Chapel Hill [Emeritus])		[11]
C-term. truncated Measles F	pCG-FΔ30	Els Verhoyen (Ecole Normale Supérieure de Lyon)		[12]
C-term. truncated Measles H	pCG-HΔ24	Els Verhoyen (Ecole Normale Supérieure de Lyon)		[12]
RD114A Env with Ampho MLV Env cytoplasmic tail	phCMV-RD114/TR	François-Loïc Cosset (Ecole Normale Supérieure de Lyon)		[13]
VSV G	pMD2.G	Didier Trono (Ecole Polytechnique Federale de Lausanne)	12259	
Rabies G	pLTR-RVG	Jakob Reiser (US FDA)	17577	[14]
Mayinga EBOV GP	pCB6-Zaire EBOV	Graham Simmons (Univ. of California, San Francisco)		[15]

Sudan Ebola GP	pCB6-Sudan EBOV	Graham Simmons (Univ. of California, San Francisco)		[15]
Reston Ebola GP	pCB6-Reston EBOV	Graham Simmons (Univ. of California, San Francisco)		[15]
Ravn MARV GP	pCAGGS-MARV	Graham Simmons (Univ. of California, San Francisco)		[16]
Bundibugyo Ebola GP	pCAGGS-BDBV	Graham Simmons (Univ. of California, San Francisco)		[17]
Taï Forest Ebola GP	pcDNA6-TAFV	Graham Simmons (Univ. of California, San Francisco)		[17]
Lloviu Ebola GP	pCAGGS-LLOV	Graham Simmons (Univ. of California, San Francisco)		[17]
Makona EBOV GP	pGL4.23 WT 2014 EBOV ΔMuc		86021	[18]
A82V Makona EBOV GP	pGL4.23 A82V 2014 EBOV ΔMuc		86022	[18]
C-term. truncated NL4.3 Env (C/O)	pcDNA-NL4.3 Env ΔCT		139285	This work
GPI anchored TVA	pCMMP-TVA800	Edward Callaway (Salk Institute)	15778	[19]
murine CAT1	pBABE-puro-mCAT1			[20]

1. Gilbert, J.M.; Bates, P.; Varmus, H.E.; White, J.M. The Receptor for the Subgroup A Avian Leukosis-Sarcoma Viruses Binds to Subgroup A but Not to Subgroup C Envelope Glycoprotein. *J. Virol.* **1994**, *68*, 5623–5628.

2. Saunders, A.A.; Ting, J.P.C.; Meisner, J.; Neuman, B.W.; Perez, M.; de la Torre, J.C.; Buchmeier, M.J. Mapping the Landscape of the Lymphocytic Choriomeningitis Virus Stable Signal Peptide Reveals Novel Functional Domains. *J. Virol.* **2007**, *81*, 5649–5657.
3. Fukushi, S.; Mizutani, T.; Saijo, M.; Matsuyama, S.; Miyajima, N.; Taguchi, F.; Itamura, S.; Kurane, I.; Morikawa, S. Vesicular Stomatitis Virus Pseudotyped with Severe Acute Respiratory Syndrome Coronavirus Spike Protein. *Journal of General Virology* 2005, *86*, 2269–2274.
4. Song, C.; Dubay, S.R.; Hunter, E. A Tyrosine Motif in the Cytoplasmic Domain of Mason-Pfizer Monkey Virus Is Essential for the Incorporation of Glycoprotein into Virions. *J. Virol.* **2003**, *77*, 5192–5200.
5. Cowan, S.; Hatziioannou, T.; Cunningham, T.; Muesing, M.A.; Gottlinger, H.G.; Bieniasz, P.D. Cellular Inhibitors with Fv1-like Activity Restrict Human and Simian Immunodeficiency Virus Tropism. *Proceedings of the National Academy of Sciences* 2002, *99*, 11914–11919.
6. Sena-Esteves, M.; Tebbets, J.C.; Steffens, S.; Crombleholme, T.; Flake, A.W. Optimized Large-Scale Production of High Titer Lentivirus Vector Pseudotypes. *J. Virol. Methods* **2004**, *122*, 131–139.
7. Robinson, L.R.; Whelan, S.P.J. Infectious Entry Pathway Mediated by the Human Endogenous Retrovirus K Envelope Protein. *J. Virol.* **2016**, *90*, 3640–3649.
8. Paterson, R.G.; Russell, C.J.; Lamb, R.A. Fusion Protein of the Paramyxovirus SV5: Destabilizing and Stabilizing Mutants of Fusion Activation. *Virology* **2000**, *270*, 17–30.
9. Hatziioannou, T.; Valsesia-Wittmann, S.; Russell, S.J.; Cosset, F.-L. Incorporation of Fowl Plague Virus Hemagglutinin into Murine Leukemia Virus Particles and Analysis of the Infectivity of the Pseudotyped Retroviruses. *Journal of Virology* 1998, *72*, 5313–5317.
10. McKay, T.; Patel, M.; Pickles, R.J.; Johnson, L.G.; Olsen, J.C. Influenza M2 Envelope Protein Augments Avian Influenza Hemagglutinin Pseudotyping of Lentiviral Vectors. *Gene Ther.* **2006**, *13*, 715–724.
11. Henkel, J.R.; Weisz, O.A. Influenza Virus M2 Protein Slows Traffic along the Secretory Pathway. pH Perturbation of Acidified Compartments Affects Early Golgi Transport Steps. *J. Biol. Chem.* **1998**, *273*, 6518–6524.
12. Moll, M.; Klenk, H.-D.; Maisner, A. Importance of the Cytoplasmic Tails of the Measles Virus Glycoproteins for Fusogenic Activity and the Generation of Recombinant Measles Viruses. *Journal of Virology* 2002, *76*, 7174–7186.
13. Sandrin, V.; Boson, B.; Salmon, P.; Gay, W.; Nègre, D.; Le Grand, R.; Trono, D.; Cosset, F.-L. Lentiviral Vectors Pseudotyped with a Modified RD114 Envelope Glycoprotein Show Increased Stability in Sera and Augmented Transduction of Primary Lymphocytes and CD34+ Cells Derived from Human and Nonhuman Primates. *Blood* **2002**, *100*, 823–832.
14. Mochizuki, H.; Schwartz, J.P.; Tanaka, K.; Brady, R.O.; Reiser, J. High-Titer Human Immunodeficiency Virus Type 1-Based Vector Systems for Gene Delivery into Nondividing Cells. *Journal of Virology* 1998, *72*, 8873–8883.
15. Simmons, G.; Wool-Lewis, R.J.; Baribaud, F.; Netter, R.C.; Bates, P. Ebola Virus Glycoproteins Induce Global Surface Protein down-Modulation and Loss of Cell Adherence. *J. Virol.* **2002**, *76*, 2518–2528.
16. Salvador, B.; Sexton, N.R.; Carrion, R.; Nunneley, J.; Patterson, J.L.; Steffen, I.; Lu, K.; Muench, M.O.; Lembo, D.; Simmons, G. Filoviruses Utilize Glycosaminoglycans for Their Attachment to Target Cells. *Journal of Virology* 2013, *87*, 3295–3304.
17. Wrensch, F.; Karsten, C.B.; Gnirß, K.; Hoffmann, M.; Lu, K.; Takada, A.; Winkler, M.; Simmons, G.; Pöhlmann, S. Interferon-Induced Transmembrane Protein–Mediated Inhibition of Host Cell Entry of Ebolaviruses. *Journal of Infectious Diseases* 2015, *212*, S210–S218.
18. Diehl, W.E.; Lin, A.E.; Grubaugh, N.D.; Carvalho, L.M.; Kim, K.; Kyawe, P.P.; McCauley, S.M.; Donnard, E.; Kucukural, A.; McDonel, P.; et al. Ebola Virus Glycoprotein with Increased Infectivity

Dominated the 2013-2016 Epidemic. *Cell* **2016**, *167*, 1088–1098.e6.
19. Wickersham, I.R.; Lyon, D.C.; Barnard, R.J.O.; Mori, T.; Finke, S.; Conzelmann, K.-K.; Young, J.A.T.; Callaway, E.M. Monosynaptic Restriction of Transsynaptic Tracing from Single, Genetically Targeted Neurons. *Neuron* **2007**, *53*, 639–647.
20. Pizzato, M. MLV Glycosylated-Gag Is an Infectivity Factor That Rescues Nef-Deficient HIV-1. *Proc. Natl. Acad. Sci. U. S. A.* **2010**, *107*, 9364–9369.
